# Book Reviews

**Published:** 1890-07

**Authors:** 


### Book Reviews.

SOME FALLACIES CONCERNING SYPHILIS. By E. L. Keyes, M. D., Consulting Physician to Bellevue, Charity and St. Elizabeth's, etc., etc. Geo. S. Davis, Detroit, Mich. 1890.

This is a small pamphlet book of seventy-one pages, which gives the author's experiences and opinions in regard to this scourge whose protean influences are so extensively experienced in this day and generation by the human family.

It is a work of considerable importance, both to the professional and the layman, since, with the one it is calculated to correct many erroneous and injurious ideas in regard to the disease; and with the other, to reduce to the minimum the certain psychical influences in disease which militate very often against rational therapeutic management. Better it would be could the laity be co-educated with the profession in the minutiæ of certain of these dyscrasiæ, and thus avoid much of the charlatanism which is moving rampant on its destructive course. Distribute the knowledge necessary to avoid the evil influence of humbuggery by spreading such works as this broadcast, and much good must result.

---

PRACTICAL ELECTRICITY IN MEDICINE AND SURGERY. By G. W. Overall, M. D., formerly Professor of Physiology, Nervous Diseases and Electro-Therapeutics in Memphis Hospital Medical College. Press of Memphis Printing Co. 1890.

This is a small book, neatly gotten up, and indicating the author to be one well posted in both the scientific and practical principles of electricity. The first part is devoted to the scientific descriptions, while electro-physiology, therapy and surgery take up the balance. As a book for ready reference we have seen none better.

---

ELECTRICITY IN THE DISEASES OF WOMEN. By G. Belton Massey, M. D., Physician to the Gynecological Department of Howard Hospital, etc., etc. Second edition. F. A. Davis, Philadelphia. 1890.

This little work is a dissertation on the practical application of electricity in the diseases of women. So clear and perspicuous is it in its descriptions that the veriest tyro may readily understand how to apply its directions, and practically carry out the idea given. On the scientific and practical description of the principles of electricity, it is complete, while its special application in female diseases is made clear and described in a very interesting manner.

The chapter on the electrical treatment of fibroid tumor of the uterus is very instructive, and is calculated to direct such attention to this subject as will lead to great improvement in its treatment.

Electricity promises to be a potent factor in the therapeusis of the future, the dawn of which is already beginning to break upon us, and no one who expects to be abreast of the times should fail to be without new issues in this line.

---

J. B. LIPPINCOTT COMPANY announce in press an important work on REGIONAL ANATOMY, in its relation to MEDICINE AND SURGERY, by George McClellan, M. D., Lecturer on Descriptive and Regional Anatomy at the Pennsylvania School of Anatomy, Professor of Anatomy at the Pennsylvania Academy of the Fine Arts; Member of the Association of American Anatomists, Academy of Natural Science, Academy of Surgery, College of Physicians, etc., of Pennsylvania.

With about 100 fac-simile illustrations, reproduced from photographs taken by the author of his own dissections. Expressly designed and prepared for this work, and colored by him after nature. To be complete in two volumes of about 250 pages each. Large quarto.

The object of the work is to convey a practical knowledge of Regional Anatomy of the entire body. The text to embrace, besides a clear description of the part in systematic order, the most recent and reliable information regarding Anatomy, in its medical and surgical relations. The illustrations are intended to verify the text and to bring before the reader the parts under consideration in as realistic a manner as possible. Vol. 1 will be ready for publication about December 1st, and the second volume is expected to appear shortly thereafter. The work will be sold by subscription only; salesmen will begin an active canvass the coming October.

